# CGR-Block: Correlated Feature Extractor and Geometric Feature Fusion for Point Cloud Analysis

**DOI:** 10.3390/s22134878

**Published:** 2022-06-28

**Authors:** Fan Wang, Yingxiang Zhao, Gang Shi, Qing Cui, Tengfei Cao, Xian Jiang, Yongjie Hou, Rujun Zhuang, Yunfei Mei

**Affiliations:** College of Information Science and Engineering, Xinjiang University, Urumqi 830017, China; xmilu0315@stu.xju.edu.cn (F.W.); zhaoyingxiang@stu.xju.edu.cn (Y.Z.); cuiqing@xju.edu.cn (Q.C.); ctfssj@stu.xju.edu.cn (T.C.); jxian1996@stu.xju.edu.cn (X.J.); 18434753056@stu.xju.edu.cn (Y.H.); zhuangrj@stu.xju.edu.cn (R.Z.); cb2@stu.xju.edu.cn (Y.M.)

**Keywords:** 3D deep learning, classification, part segmentation, point cloud

## Abstract

Point cloud processing based on deep learning is developing rapidly. However, previous networks failed to simultaneously extract inter-feature interaction and geometric information. In this paper, we propose a novel point cloud analysis module, CGR-block, which mainly uses two units to learn point cloud features: correlated feature extractor and geometric feature fusion. CGR-block provides an efficient method for extracting geometric pattern tokens and deep information interaction of point features on disordered 3D point clouds. In addition, we also introduce a residual mapping branch inside each CGR-block module for the further improvement of the network performance. We construct our classification and segmentation network with CGR-block as the basic module to extract features hierarchically from the original point cloud. The overall accuracy of our network on the ModelNet40 and ScanObjectNN benchmarks achieves 94.1% and 83.5%, respectively, and the instance mIoU on the ShapeNet-Part benchmark also achieves 85.5%, proving the superiority of our method.

## 1. Introduction

Object classification and semantic segmentation, as the basic technologies for point cloud processing and analysis, have become the research focus in the fields of autonomous driving, robotics, high-precision maps, SLAM, etc. Point cloud processing can be divided into traditional methods and deep learning methods. Nowadays, deep learning methods occupy an absolute dominant position in the field of object detection and semantic segmentation of 2D images. However, due to the characteristics of the disorder, density inconsistency, nonstructure, and incomplete information of 3D point cloud data, object classification and semantic segmentation of point clouds are full of challenges, and the related models based on deep learning methods are far less mature than the 2D image field.

The deep learning processing of 2D images mainly relies on the convolution operator. The convolution operator samples a center on a uniform grid with a fixed step size, and then aggregates the features around the point according to the pre-set convolution kernel size. The receptive field is gradually enlarged by stacking multiple convolutional layers, while expanding the number of channels. Inspired by the 2D convolution operator, many previous works [1,2,3] have attempted to convert point clouds from irregular data to regular data. This type of method first captures 2D snapshots of 3D shapes from different perspectives, then uses traditional convolution to extract features from each image, and finally applies pooling layers and fully connected layers to aggregate images from different perspectives to obtain the final semantic features.

To directly process raw point data, PointNet [4] learns each point independently and collects the global feature representation of the points. This work is pioneering, but this design ignores the local geometric information of point clouds, which is an important factor for image CNNs to extract high-level visual representations. PointNet++ [5] extends PointNet and constructs a basic architecture of sampling, grouping, feature extraction, and aggregation, which provides a basic reference for the subsequent architectural design of point cloud analysis networks. Most of the mainstream networks are based on this, but they are different in terms of feature mining and processing. These can be mainly classified into the following three categories:

(1) Perform max-pooling functions to aggregate the features of neighbor points indiscriminately (e.g., PointNet++). Although this scheme is currently the most widely adopted approach for point cloud analysis networks, and has the advantage of cheap computational complexity and a positive impact on inference speed, the method does not distinguish semantic differences between features.
(2) Construct a multilayer perceptron (MLP) that takes a set of neighbor point features as input, outputs a set of weights, and then uses the weights to perform a weighted summation of the neighbor point features (e.g., RandLA-Net [6]). However, a simple MLP has difficulty learning a meaningful set of weights, and using the learned weights to rescale the features yields no observable improvement over undifferentiated max-pooling.
(3) Use the self-attention mechanism to capture the long-distance interaction between point features in a purely data-driven and learning-based way, and adjust the features adaptively (e.g., PointTransformer [7], PCT [8]). Although the result of this scheme is remarkable, since the computational complexity and memory consumption of the self-attention mechanism are $O(N^{2})$, where *N* is the input point number, the naive self-attention is not suitable for processing point clouds.

Based on the above analysis, we designed a correlated feature extractor unit. Different from previous work, this unit assigns weights to each neighbor point feature mainly based on the Euclidean distance of the neighbors from its center point, and the details of this unit are in Section 3.3. This process is heuristic and does not have an expensive learning process, in which the distance matrix is calculated only once in the previous KNN process. This unit has strong interpretability for the scaling process of features.

In addition, in the process of feature extraction, the previous work either over-focused on the mining of deep semantic information while ignoring local geometric information (e.g., direction, distance), or over-relied on local geometric information that local geometric features increase with the depth of the network layers, resulting in encoding too much redundant information, therefore, a geometric feature fusion unit is proposed in this paper, and the difference of the proposed unit is that the geometric feature fusion unit only outputs a fixed-dimensional and compact token, which encodes key information such as the direction and distance of neighbors relative to the center point, and the computational complexity generated by this unit is negligible with respect to the entire learning process, but produces a considerable contribution to the improvement of the final results.

In this paper, the encoder module is mainly built based on the abovementioned unit, called CGR-block, i.e., the Correlated feature extractor unit is used to extract deep semantic features of point clouds and the interactions between their neighbor points, and the Geometric feature fusion unit is used to output a compact geometric pattern token of the current stage, which is embedded in the feature extractor and effectively fused. In addition, a Residual mapping branch [9] is used to mitigate network degradation before neighbor point feature aggregation, which enables the entire network to easily expand to a deeper level.

Using the CGR-block as a universal encoder, which is integrated into the classic point cloud analysis pipeline without changing the network configuration, the results of the CGR-block are excellent even on simple networks. We test the performance of CGR-block on the two most popular object classification benchmarks, ModelNet40 [10] and ScanObjectNN [11], and also test the performance of part segmentation on the ShapeNet-Part benchmark [12]. Experiments show that the network constructed by CGR-block achieves comparable or better performance than current state-of-the-art methods on multiple challenging benchmarks and tasks.

The main contributions of our work are as follows:

- The proposed CGR-block can simultaneously extract and fuse abstract semantic features and local geometric tokens of the point cloud, and it can serve as the basic module to construct the network for point cloud analysis.
- The proposed correlated feature extractor unit can mine inter-point interaction information in a heuristic way and extract features efficiently.
- The proposed geometric feature fusion unit generates a compact geometric pattern token at each stage of feature extraction and fuses it into the deep semantic feature extraction process, which provides considerable contribution with a weak overhead.
- CGR-Net performs multiple experiments on the point cloud classification and part segmentation tasks to verify that the network constructed by the proposed CGR-block can achieve or outperform state-of-the-art approaches.

## 2. Related Work

### 2.1. Multiple-View-Based and Voxel-Based Methods

The representative work based on multiple views MVCNNs was first proposed by Su et al. [1], which captures 2D views of 3D objects under different perspectives, performs image feature extraction for each view, and aggregates the images from different perspectives through the max-pooling and fully connected layer to obtain the final global features. Although an MVCNN can integrate the image features of different views well, it does not utilize the fine-grained geometric information of each image effectively and cannot select the views dynamically. Feng et al. proposed a group-view convolutional neural network (GVCNN) [13] based on MVCNNs, which groups features extracted from different images and can effectively utilize the relationship between multiple images. However, such multi-view-based methods will inevitably lose a large amount of critical geometric information. VoxNet proposed by Maturana et al. [14] alleviates the problem of geometric information loss to some extent. However, compared to 2D images, voxelization has more computational overhead due to the addition of one dimension, and the computational complexity limits the improvement of voxel resolution [15].

Therefore, multi-view-based and voxel-based methods are often seen as a transitional stage, and the scheme of directly consuming unordered point sets as inputs to extract features is the general trend.

### 2.2. Discrete Point-Based Methods

Qi et al. proposed PointNet [4], which directly takes point clouds as its input and achieves permutation invariance with a max-pooling function. Specifically, PointNet learns pointwise features independently with several shared MLP layers and extracts global features with a max-pooling layer. However, PointNet independent extraction cannot obtain fine-grained local feature information, and cannot fully consider the interaction between adjacent points. PointNet++ [5] is an extension of PointNet, which implements a set abstraction (SA) module similar to the convolution operation to aggregate local area features hierarchically, but PointNet++ still does not mine the relationship between the center point and its neighbor points (e.g., directionality). The learning of local features is still insufficient, and the interaction information between points is still not fully utilized.

However, the pipeline proposed by PointNet++ provides a basic reference for the subsequent point cloud analysis network, and the idea of layer-by-layer sampling and aggregation is widely used in subsequent work. Wang et al. improved PointNet++ and proposed a dynamic graph convolutional neural network, DGCNN [16]. [DGCNN constructs a local neighbors map and uses the edge convolution (EdgeConv) operator to extract the features of the center point and the edge vector of the neighbor points to obtain the local pattern. EdgeConv considers the distance between center points and neighbors, but ignores information such as vector direction between adjacent points. RandLA-Net [6] uses local spatial encoding (LocSE) to encode the geometric patterns of local neighbors, but the output dimension of LocSE increases with the number of stacking layers of modules, which causes it to encode too much redundant information.

In addition, PCT [8] introduces self-attention to point cloud processing to capture long-distance point-to-point interactions. ECC [17] and DPAM [18] use graph neural networks or graph convolution to process point clouds. So-net [19] simulates the spatial distribution of point clouds by constructing a self-organizing map (SOM). PointCNN [20] learns the X-transform convolution operator to adaptively arrange local point cloud features. Shape Context Net (SCN) [21] designs shape context bins to capture the local geometry of point clouds. Although the results of these methods may be satisfactory, these methods fail to enjoy the advantages of highly optimized convolution and MLP computational efficiency, generally facing high computational costs, and they are difficult to integrate with existing pipelines.

Our network follows the hierarchical design concept of PointNet++. The entire network mainly consists of shared MLPs without relying on any expensive operations such as graph construction and kernelization.

## 3. Methods

This section describes the details of the geometric feature fusion and correlated feature extractor unit, as well as the detailed structure of the CGR-block. Then, an end-to-end network built based on CGR-block is proposed for point cloud classification and segmentation tasks.

### 3.1. Overview

In most hierarchical network architectures, the input (e.g., coordinates x–y–z, surface normal information, R–G–B, intensity of point clouds) is first mapped to an abstract feature space through a point embedding layer. Then, they use the sampling, grouping, and aggregation to expand the receptive field layer by layer, increase the feature dimension, and finally obtain a global feature descriptor. However, most of the networks lack attention to the local geometric structure in the process of improving the dimension of point features, indiscriminately aggregate the features of the center point and its neighbor points at each stage, and lack the extraction of the correlation between point features. Therefore, the important features and key information are not effectively preserved. In addition, the previous network architecture is difficult to extend to a deeper level, which leads to the limited expressive ability of the entire network and it cannot fully extract deeper-level features.

To this end, we design CGR-block, a general module that can perform differentiated aggregation on the neighbor point features, and fuse the geometric pattern into the deep semantic features synchronously in each stage of feature extraction. In addition, the residual mapping branch is introduced in the CGR-block, so that the module can integrate the feature information of the previous stage.

### 3.2. Farthest Point Sampling (FPS) and K-Nearest Neighbors Grouping (KNN)

Given a set of input coordinate points and their corresponding latent features, denoted as $P^{in}=\left\{p^{i}|i=1,2,3,\cdots ,N^{in}\right\}\in R^{N^{in}\times 3}$ and $F^{in}=\left\{f_{i}|i=1,2,3,\cdots ,N^{in}\right\}\in R^{N^{in}\times D}$, respectively, we use the widely adopted farthest point sampling (FPS) algorithm, sample $N^{s}$ center points from the input point set $P^{in}$, and use the K-nearest neighbors (KNN) algorithm to select k neighbors around the center points as $N^{s}$ local patches. The point coordinates and corresponding features of the *i*-th patch are denoted as $\left\{p_{i,1},p_{i,2},p_{i,3},\cdots ,p_{i,k}\right\}\in R^{k\times 3}$ and $\left\{f_{i,1},f_{i,2},f_{i,3},\cdots ,f_{i,k}\right\}\in R^{k\times D}$. In addition, we maintain a distance matrix $A_{i}=\left\{a_{i,k}\right\}\in R^{k\times 1}$ when looking for K-nearest neighbors ($a_{i,k}$ represents the distance from the *k*-th neighbor point to its center point), and output the distance matrix $A_{i}$ together with the local patches. The matrix $A_{i}$ will be further processed in the next section.

### 3.3. Correlated Feature Extractor

We do not indiscriminately aggregate the features of each center point and its neighbor points, but give different weights to each neighbor point. The weight calculation idea is based on such a geometric prior: in the space of the point cloud, the closer the Euclidean distance is, the closer the semantic relationship between them is. Intuitively, the closer the points are in the point cloud space, the more likely they are the same object or the same part of the same object. Therefore, we assign higher weights to the features of neighbors whose Euclidean distance is closer to its corresponding center point and lower weights to those that are farther from the sampled center. In the implementation, the distance matrix $A_{i}$ is first normalized, and then its reciprocal is taken to construct the inverse distance weight matrix ${\tilde{A}}_{i}$. Given the feature set $F_{i}=\left\{f_{i,k}\right\}\in R^{k\times D}$ of the neighbor points, the details of recalculating the features of each neighbor point can be expressed as follows:

(1) $$a_{i,j}=\left|\right|p_{i,j}-p_{i}\left|\right|,0⩽j⩽k-1$$

(2) $$A_{i}=\left\{a_{i,1},a_{i,2},a_{i,3},\cdots ,a_{i,k}\right\}\in R^{k\times 1}$$

(3) $${\tilde{A}}_{i}=\frac{1}{\mathrm{softmax}(A_{i})}$$

(4) $${\tilde{F}}_{i}=F_{i}⊙{\tilde{A}}_{i}$$

where ⊙ is the elementwise (Hadamard) product. To improve the stability of the calculation, the affinity matrix $A_{i}$ is normalized using the softmax function. The weight ${\tilde{a}}_{i,j}$ of the *j*-th neighbor of the center point is a monotonically decreasing function of the distance from the point to its center point. The feature after adjusting the weight is denoted as ${\tilde{F}}_{i}$. In addition, the feature of the center point sampled by the FPS algorithm summarizes a representative feature of the current local patch. Thus, the center point feature $f_{i}$ is repeated k times, and then concatenated with the corresponding neighbor point features ${\tilde{F}}_{i}$. The combined feature dimension naturally becomes twice the input feature, and then we apply an LBR (combining Linear, BatchNorm, and ReLU layers) element to the feature map for preliminary integration, and the output is denoted as ${\tilde{F}}_{i}{}^{{}^{\prime }}$.

**Figure 1:**
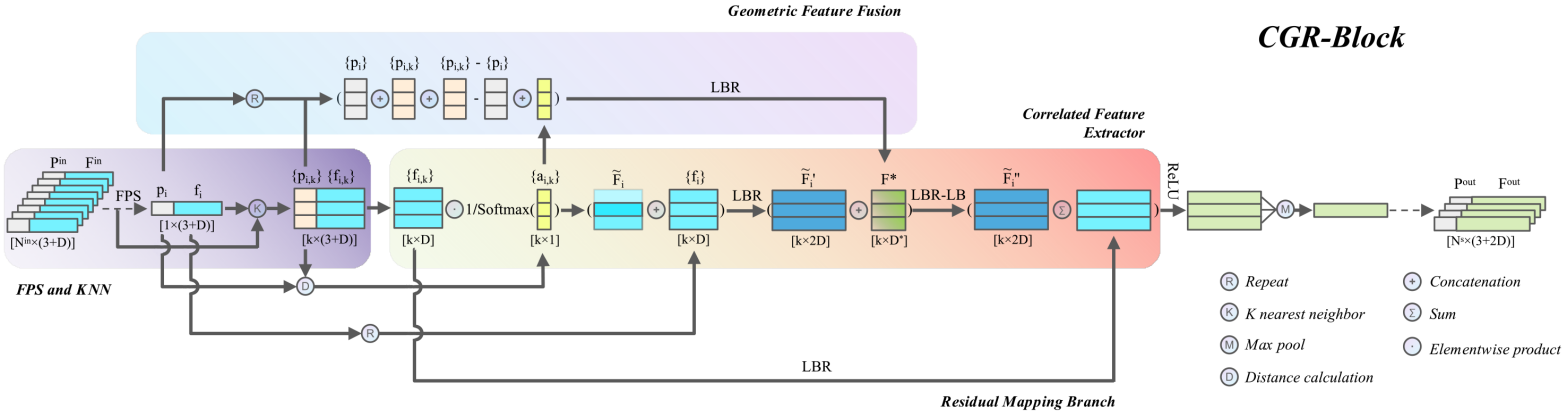
The structure of CGR-block. The CGR-block consists of an FPS and KNN unit (**middle left**), a correlated feature extractor (**middle right**), a geometric feature fusion (**top**), and a residual mapping branch (**bottom**). LBR combines Linear, BatchNorm, and ReLU layers. The top and bottom of each tensor are marked with a symbol and shape, respectively.

Note that all of our operations so far have only learned abstract point features, but have not integrated local geometric relations at each stage. However, the encoding and embedding of local geometric relations are crucial to the point cloud understanding task. To this end, a geometric feature fusion unit is designed in the CGR-block to produce a compact token $F^{*}$ of the local geometric pattern. We fuse $F^{*}$ into ${\tilde{F}}_{i}^{{}^{\prime }}$, and apply the LBR element twice to the feature vector ${\tilde{F}}_{i}^{{}^{\prime }}⊕F^{*}\in R^{k\times (2D+D^{*})}$ embedded in the geometric relationship token to extract the more expressive geometric and deep semantic information of the current stage, as shown in Figure 1. More details of the geometric feature fusion unit will be presented in Section 3.4.

### 3.4. Geometric Feature Fusion

Since the correlated feature extractor unit extracts high-dimensional semantic features for points and the interaction information between them, the attention of local fine-grained geometric information is ignored. However, information such as relative position, orientation, and distance is crucial in point cloud understanding tasks. Inspired by position embeddings [22] in NLP tasks, we propose a geometric feature fusion unit that computes the relative position, direction, distance, and other geometric information of the neighbor points relative to the center in each stage [6]. The calculation process is as follows:

(5) $$F^{*}=LBR(p_{i}⊕p_{i,k}⊕\left(p_{i,k}-p_{i}\right)⊕\left|\right|p_{i,k}-p_{i}||),$$

where $p_{i}$ and $p_{i,k}$ represent coordinates of the center point and its *k* neighboring points in the *i*-th local patch, respectively, ⊕ denotes the concatenation operator, and ‖·‖ denotes calculating the Euclidean distance, the LBR(·) element represents a nonlinear mapping consisting of Linear, BatchNorm and ReLU layers.

The output of this process $F^{*}$ is embedded in the preliminary integrated feature ${\tilde{F}}_{i}^{{}^{\prime }}$. Our geometric feature fusion unit is similar to the LocSE module in RandLA-Net [6]. The difference is that the output dimension of the geometric feature fusion unit does not increase with the stacking of modules, but it outputs a compact geometric token with a fixed dimension in each stage. In the implementation, we set the dimension $D^{*}=128$), so as to avoid encoding too much redundant information with the geometric feature fusion unit during the stacking of modules, and to maintain efficient computation. The geometric feature fusion enables the network to be always aware of local geometric patterns at each stage, which ultimately benefits the entire network to effectively learn complex local structures.

### 3.5. CGR-Block

CGR-block was built using the units introduced in Section 3.2, Section 3.3 and Section 3.4. As shown in Figure 1, the local patches obtained by farthest point sampling (FPS) and K-nearest neighbor grouping (KNN) are input to the geometric feature fusion and the correlation feature extraction unit. In the correlated feature extractor unit, the neighbor point features are rescaled using the weight matrix ${\tilde{A}}_{i}$, whose rescaled features are denoted as ${\tilde{F}}_{i}$, and then it is concatenated with the center point feature $\left\{f_{i}\right\}$. This process can be formulated as ${\tilde{F}}_{i}⊕repeat\left(f_{i},k\right)$, where the function repeat(·) copies the center point feature $f_{i}$*k* times. Next, an LBR element is applied to the concatenated feature ${\tilde{F}}_{i}⊕repeat\left(f_{i},k\right)$ for preliminary integration, and the output features are denoted as ${\tilde{F}}_{i}^{{}^{\prime }}\in R^{k\times 2D}$, where ${\tilde{F}}_{i}^{{}^{\prime }}=LBR\left({\tilde{F}}_{i}⊕repeat\left(f_{i},k\right)\right)$. Then, the geometric token $F^{*}$ output by the geometric feature fusion unit is concatenated with ${\tilde{F}}_{i}^{{}^{\prime }}$, and the concatenated feature vector ${\tilde{F}}_{i}^{{}^{\prime }}⊕F^{*}\in R^{k\times (2D+D^{*})}$ is further fused using LBR×2 element to extract more advanced semantic features. This process can be formulated as $ReLU(LBR-LB\left(F_{i}^{{}^{\prime }}⊕F^{*}\right)+LBR\left(\left\{f_{i,k}\right\}\right))$, where LBR(·) and LBR−LB(·) elements represent nonlinear mappings. Note that before the last ReLU function of the LBR×2 element, a residual mapping branch is used to aggregate the features of the previous stage into the current stage. The implementation of the residual mapping branch is based on an LBR element for mapping the input features to the same dimension as ${\tilde{F}}_{i}^{{}^{\prime \prime }}$. Finally, a max-pooling function is used to aggregate the features of each patch. The whole process can be summarized as:

(6) $$F^{out}=MAX\left(ReLU\left(LBR-LB\left(LBR\left({\tilde{F}}_{i}⊕repeat\left(f_{i},k\right)\right)⊕F^{*}\right)+LBR\left(\left\{f_{i,k}\right\}\right)\right)\right),$$

where MAX(·) represents the max-pooling aggregation, and $F^{out}$ is the final output of the CGR-block, representing the features of each local patch.

### 3.6. Network Architecture

**Figure 2:**
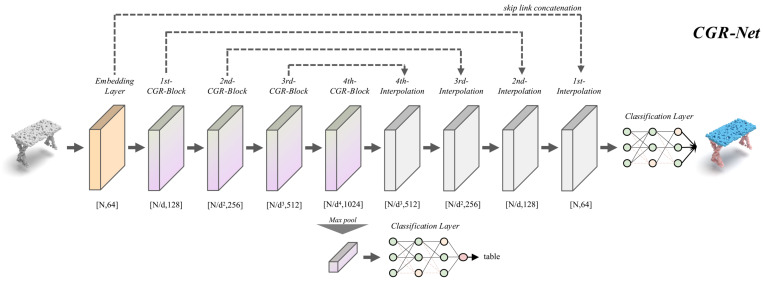
The structure of point cloud classification and segmentation networks constructed by the proposed CGR-block. The number below the module represents the output shape of the current module. “*d*” denotes the decimation ratio of each stage.

The network architecture of CGR-Net is shown in Figure 2. Following the traditional hierarchical pipeline, CGR-Net extracts features from local to global. We set up a point embedding layer and a classification layer at the front end and the back end of the network. The point embedding layer is constructed of an LBR element, and the classification layer is constructed using an LBR with dropout (LBRD) element. In the object classification network, we map the output of the last CGR-block to the same size as the number of categories using a classification layer. In the semantic segmentation network, our upsampling strategy adopts trilinear interpolation similar to PointNet++ [5], and propagates the features to the original points stage by stage through skip link concatenation [23]. Finally, we use the classification layer to output the pointwise category labels.

It should be noted that we set the decimation ratio d=2 in the classification network, and d=4 in the part segmentation network.

## 4. Results

To validate the performance of CGR-block, we evaluate the performance of our model on several of the most popular benchmarks. For the object classification task, we adopt ModelNet40 [10] and ScanObjectNN [11] to evaluate the performance of CGR-block on synthetic and real-world datasets, respectively. We also adopt the ShapeNet-Part benchmark [12] to test the performance of our part segmentation task. In addition to that, this section also reports the results of ablation experiments to demonstrate the effectiveness of the components of the CGR-block and to illustrate the choice of some important parameters.

### 4.1. Classification on ModelNet40

The ModelNet40 [10] dataset contains 12,311 synthetic object from 40 categories. We follow the official dataset split, 9843 objects for training and 2468 objects for testing. In terms of data sampling and augmentation, the normalized 1024 points and the corresponding normal vectors are sampled uniformly from each object, and the data are enhanced by adding Gaussian jitter, point random drop, and random scale scaling to each coordinate point of the object, where the mean of the Gaussian jitter is 0 and the standard deviation of it is 0.05; the scale of random scaling is between 0.8 and 1.25; the probability of each point dropping out ranges from 0 to 0.875. The Adam optimizer with an initial learning rate of 0.001 and a weight decay of 0.0001 is used. In addition, the learning rate of each parameter group decays by 0.8 every 20 epochs. CGR-Net was trained with cross-entropy loss. A batch size of 32 and a maximum epoch of 300 are set for training.

As shown in Table 1, the point cloud classification network composed of CGR-blocks proposed in this paper is far better than the network that only extracts point features. The OA of CGR-Net is 2.2% higher than that of PointNet++ [5]. At the same time, the performance of CGR-Net is better than that of PCT [8] and Point Transformer [7], which use expensive self-attention algorithms to mine neighbor point associations. Even the OA of our network is 0.5% higher than that of RS-CNN [24] and GDANet [25] using a voting strategy. The final score on our test set does not use any voting strategy and post-processing, including experimental results on two other benchmarks. The experimental results show that CGR-Net is still better than other networks when it is mainly based on the general operator of the existing framework.

### 4.2. Classification on ScanObjectNN

Although the proposed network achieved such impressive results on the ModelNet40 benchmark [10], however, the experimental results on synthetic data usually cannot represent the generalization performance of the network in the real world. When the objects are framed with real-world settings, the object classification is still a challenging task. To this end, we also test the ScanObjectNN benchmark [11], a recently released real-world point cloud object classification dataset, which has over 15,000 objects in only 15 categories. It is practically more challenging due to the background complexity, object partiality, and different deformation variants, and its classification difficulty is much higher than the idealized data in ModelNet40. CGR-Net was trained and tested on the hardest variant (PB_T50_RS). Our loss function, optimizer, learning rate evolution scheduler, and data augmentation scheme all keep the same settings as in Section 4.1, and the only difference is that we only use x–y–z coordinates as input to the embedding layer.

Table 2 shows our results on the ScanObjectNN benchmark compared to other networks. Note that our class-averaged accuracy (mAcc) achieves much higher results than other similar models. Furthermore, our method achieves the smallest gap between class-averaged accuracy and overall accuracy. This result indicates that our method is not biased towards a particular class, showing fairly good robustness.

### 4.3. Part Segmentation on the ShapeNet-Part

Compared with object classification, part segmentation is a fine-grained recognition task that predicts the part category label (e.g., chair leg, cup handle) for each point of the input point cloud object. The ShapeNet-Part dataset [12] contains 16,880 shapes from 16 shape categories, with a total of 50 different parts, and most objects are annotated with 2 to 4 parts, with a 14,006 training and 2874 testing split.

In the CGR-Net configuration, each instance is sampled with 2048 points and their normal vectors as input to the embedding layer. In terms of network configuration, except that no additional equispaced learning rate adjustment is used, the loss function, optimizer configuration, and data augmentation scheme are kept consistent with the classification network. The results are shown in Table 3.

**Figure 3:**
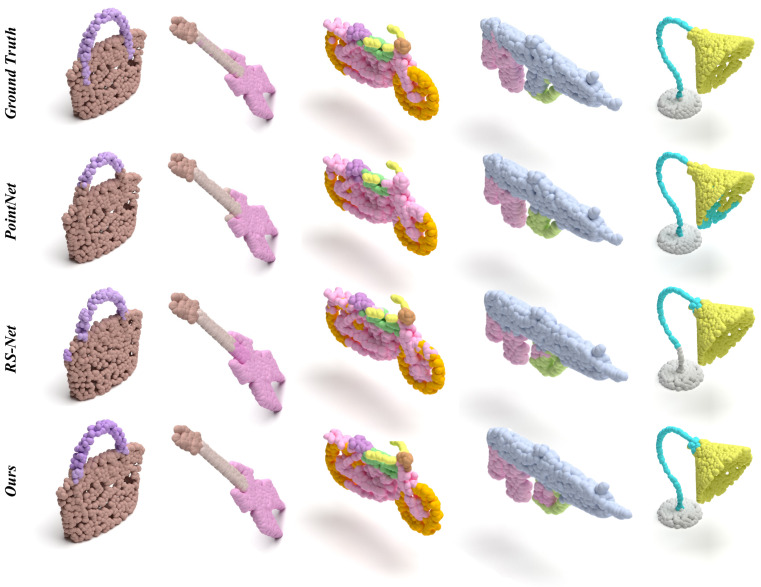
Segmentations from ground truth, PointNet, RS-Net, and ours.

Figure 3 shows the comparison between PointNet [4], RS-Net [40], our method, and ground truth. As shown in the figure, our method also performs well on the part segmentation task.

### 4.4. Ablation Studies

#### 4.4.1. The Validity of the Components of CGR-Block

CGR-block is mainly constructed of a correlated feature extractor, geometric feature fusion, and residual mapping branch. To verify that combining the above three components can lead to better performance, we build the following four networks. The first network simplifies the correlated feature extractor unit (we cancel the weight calculation of neighbor point features in this unit and simply combine the center point and neighbor point features). The second network removes the geometric feature fusion unit, and the third network preserves the geometric feature fusion and correlated feature extractor unit, but removes the residual mapping branch as a bridge to add the features of the previous stage. The last network consists of a complete CGR-block, in which the above three units work together. We evaluate the above four networks on ModelNet40 [10], and their performances are listed in Table 4. It can be seen that the class-averaged accuracy (mAcc) of the fourth network is higher than that of the other three networks by 0.3%, 1.7%, 0.1%, respectively. In terms of overall accuracy (OA), that of the fourth network is higher than that of the other three networks by 0.4%, 1.9%, 1.0%, respectively.

The experimental results show that each component of the CGR-block has an important impact on the performance of the network. In addition, the biggest impact on the network is the removal of the geometric feature fusion unit, which again confirms that the attention to fine-grained geometric structure has an important impact on the network learning the complex geometric structure of point clouds.

#### 4.4.2. The Output Dimension of the Geometric Feature Fusion Unit

The output dimension of the geometric feature fusion unit affects the effectiveness of the geometric pattern encoding and the model complexity of the network. Intuitively, the larger the output dimension is, the more information it can encode. However, the statistics in Table 5 show that the high output dimension of the geometric feature fusion unit makes it encode too much redundant information, and will overwrite the information extracted by the correlated feature extractor unit during the fusion process. In view of this, we experiment with different output dimensions of the geometric feature fusion unit, of which the $D^{*}$ of the classification network is set to 16, 32, 64, 128, 256, respectively, and experiment on the ModelNet40 benchmark [10]. The results are shown in Table 5.

It can be seen that the size of the model parameters and FLOPs/sample increase with $D^{*}$, but the accuracy is not positively correlated with the size of $D^{*}$. The experimental results show that if $D^{*}$ is too small, it cannot fully encode geometric information, while if it is too large, the network will be saturated and the performance will be reduced. Therefore, CGR-Net sets the output dimension $D^{*}=128$.

## 5. Conclusions

In this paper, we propose a simple-structured learnable module CGR-block, which can be easily integrated into existing hierarchical point cloud understanding networks. The module is mainly composed of a correlated feature extractor, geometric feature fusion, and residual mapping branch. The correlated feature extractor module integrates center point features, adjusts the weight of neighbor point features, and applies a multi-stage shared MLP to extract features. Geometric feature fusion produces a compact representation of the current geometric pattern and fuses it into the associated feature extraction process. In addition, a large-span residual mapping branch is introduced in the CGR-block to reduce the degradation in deep neural networks. Using CGR-block as the basic module to build the end-to-end network CGR-Net, and conducting experiments on multiple benchmarks, the proposed CGR-Net achieves state-of-the-art performances on multiple benchmarks.

## Figures and Tables

**Table 1:** Results on the ModelNet40 shape classification. The evaluation metrics are the mean accuracy within each category (mAcc) and the overall accuracy (OA) over all classes (P: point, N: normal, “*”: voting, “-”: unknown). The bolded text indicates the best results.

Methods	Inputs	#Points	mAcc (%)	OA (%)
PointNet [4]	P	1k	86.0	89.2
PointNet++ [5]	P + N	1k	-	91.9
SpiderCNN [26]	P + N	1k	-	92.4
PointCNN [20]	P	1k	88.1	92.5
PointConv [27]	P + N	1k	-	92.5
A-CNN [28]	P + N	1k	90.3	92.6
Point Trans. (Engel et al., 2020) [29]	P	1k	-	92.8
DGCNN [16]	P	1k	90.2	92.9
MLMSPT [30]	P	1k	-	92.9
RS-CNN [24]	P	1k	-	92.9
PointASNL [31]	P	1k	-	92.9
KPConv [32]	P	7k	-	92.9
PCT [8]	P	1k	-	93.2
PosPool [33]	P	5k	-	93.2
DensePoint [34]	P	1k	-	93.2
PointASNL [31]	P + N	1k	-	93.2
RS-CNN * [24]	P	1k	-	93.6
Point Trans. (Zhao et al., 2021) [7]	P	1k	90.6	93.7
GDANet * [25]	P	1k	-	93.8
**Ours**	P + N	1k	**91.9**	**94.1**

**Table 2:** Results on the ScanObjectNN object classification. The bolded text indicates the best results.

Methods	mAcc (%)	OA (%)
3DmFV [35]	58.1	63.0
PointNet [4]	63.4	68.2
SpiderCNN [26]	69.8	73.7
PointNet++ [5]	75.4	77.9
DGCNN [16]	73.6	78.1
PointCNN [20]	75.1	78.5
BGA-DGCNN [11]	75.7	79.7
BGA-PN++ [11]	77.5	80.2
DRNet [36]	78.0	80.3
GBNet [37]	77.8	80.5
SimpleView [38]	-	80.5 ± 0.3
PRANet [39]	79.1	82.1
**Ours**	**82.7**	**83.5**

**Table 3:** Shape part segmentation results on ShapeNet-Part benchmark. The bolded text indicates the best results.

Methods	Class mIoU	Ins-Tance mIoU	Airplane	Bag	Cap	Car	Chair	Earphone	Guitar	Knife	Lamp	Laptop	Motorbike	Mug	Pistol	Rocket	Skateboard	Table
Kd-Net [41]	77.4	82.3	80.1	74.6	74.3	70.3	88.6	73.5	90.2	87.2	81.0	94.9	57.4	86.7	78.1	51.8	69.9	80.3
PointNet [4]	80.4	83.7	83.4	78.7	82.5	74.9	89.6	73.0	91.5	85.9	80.8	95.3	65.2	93.0	81.2	57.9	72.8	80.6
SCN [21]	81.8	84.6	83.8	80.8	83.5	79.3	90.5	69.8	91.7	86.5	82.9	96.0	69.2	93.8	82.5	**62.9**	74.4	80.8
SPLATNet [42]	82.0	84.6	81.9	83.9	**88.6**	**79.5**	90.1	73.5	91.3	84.7	84.5	**96.3**	69.7	95.0	81.7	59.2	70.4	81.3
SO-Net [19]	80.8	84.6	81.9	83.5	84.8	78.1	90.8	72.2	90.1	83.6	82.3	95.2	69.3	94.2	80.0	51.6	72.1	82.6
SyncCNN [43]	82.0	84.7	81.6	81.7	81.9	75.2	90.2	74.9	**93.0**	86.1	84.7	95.6	66.7	92.7	81.6	60.6	**82.9**	82.1
KCNet [44]	82.2	84.7	82.8	81.5	86.4	77.6	90.3	**76.8**	91.0	87.2	84.5	95.5	69.2	94.4	81.6	60.1	75.2	81.3
RS-Net [40]	81.4	84.9	82.7	**86.4**	84.1	78.2	90.4	69.3	91.4	87.0	83.5	95.4	66.0	92.6	81.8	56.1	75.8	82.2
DGCNN [16]	82.3	85.1	**84.2**	83.7	84.4	77.1	**90.9**	78.5	91.5	**87.3**	82.9	96.0	67.8	93.3	82.6	59.7	75.5	82.0
PCNN [45]	81.8	85.1	82.4	80.1	85.5	**79.5**	90.8	73.2	91.3	86.0	**85.0**	95.7	73.2	**94.8**	**83.3**	51.0	75.0	81.8
PointNet++ [5]	81.9	85.1	82.4	79.0	87.7	77.3	90.8	71.8	91.0	85.9	83.7	95.3	71.6	94.1	81.3	58.7	76.4	82.6
SpiderCNN [26]	82.4	85.3	83.5	81.0	87.2	77.5	90.7	**76.8**	91.1	**87.3**	83.3	95.8	70.2	93.5	82.7	59.7	75.8	**82.8**
**Ours**	**82.8**	**85.5**	82.4	79.7	87.7	79.4	90.4	76.2	91.2	85.7	84.3	95.8	**75.3**	94.7	81.5	61.4	76.6	82.2

**Table 4:** The classification performance of ablated network on ModelNet40. The bolded text indicates the best results.

Ablations	mAcc (%)	OA (%)
(1) Simplify correlated feature extractor	91.6	93.7
(2) Remove geometric feature fusion	90.2	92.2
(3) Remove shortcut	91.8	93.1
**(4) The full network**	**91.9**	**94.1**

**Table 5:** Result and model complexity comparison with different dimensions of geometric feature fusion. The bolded text indicates the best results.

$D^{*}$	16	32	64	128	256
mAcc (%)	91.0	91.2	91.6	**91.9**	91.7
OA (%)	93.5	93.6	93.9	**94.1**	93.5
#params (M)	**5.66**	5.75	5.95	6.37	7.31
#FLOPs/sample (M)	**1461.3**	1504.7	1596.2	1798.2	2277.6

## Data Availability

The datasets used in this paper are publicly available, including ModelNet40, ScanObjectNN, and ShapeNet-Part.
